# Washcoat Nanoparticle-Driven Microcrack Enlargement in a Coated Catalyst

**DOI:** 10.1021/acsaenm.5c00142

**Published:** 2025-05-12

**Authors:** Siddhant Naudiyal, Mark S’ari, Martha Briceno de Gutierrez, Paul Bowen, Mark John H. Simmons, E. Hugh Stitt, Aswani Mogalicherla

**Affiliations:** † School of Chemical Engineering, 1724 University of Birmingham, Edgbaston Campus, Birmingham B15 2TT, England, U.K.; ‡ Johnson Matthey Technology Centre, 41975 Johnson Matthey plc, Belasis Avenue, Stockton-on-Tees, Billingham TS23 1LH, England, U.K.; § Johnson Matthey Technology Centre, 41975 Johnson Matthey plc, Blounts Ct Rd, Sonning Common, Reading RG4 9NH, England, U.K.; ∥ School of Metallurgy and Materials, 1724 University of Birmingham, Edgbaston Campus, Birmingham B15 2TT, England, U.K.

**Keywords:** impulse excitation technique, microcracks, TEM, FIB-SEM, SEM, 3-point bend test, aluminum titanate

## Abstract

Nanoparticles are avoided in coating particulate filters made from microcracked ceramics like aluminum titanate (AT) to prevent thermal stress damage during manufacturing despite limited understanding of the process. This study, first in this field, investigated the effect of fines penetration (*D*_50_: 21 nm) from washcoating on the thermomechanical properties and long-term durability of a microcracked AT honeycomb filter substrate. Through a combination of bespoke SEM, FIB-SEM, and TEM methodologies developed, visual evidence of nanoparticles from the washcoat penetrating microcracks in the substrate has been obtained. The impact of this penetration upon the substrate's mechanical properties has been successfully evaluated using dynamic stiffness measurements through a high-temperature impulse excitation technique (IET), providing an alternative detection methodology to time-consuming and expensive microscopy measurements for product development. The IET measurements revealed that the fines penetration hinders the closing of microcracks, leading to their enlargement. These microstructural changes are not indicated by strength testing, as the coating increased the substrate strength. However, IET experiments confirmed that the effect of the penetration has been shown to stabilize after three thermal cycles, ensuring the structural stability of these substrates even after applying a coating with nanoparticles.

## Graphical abstract

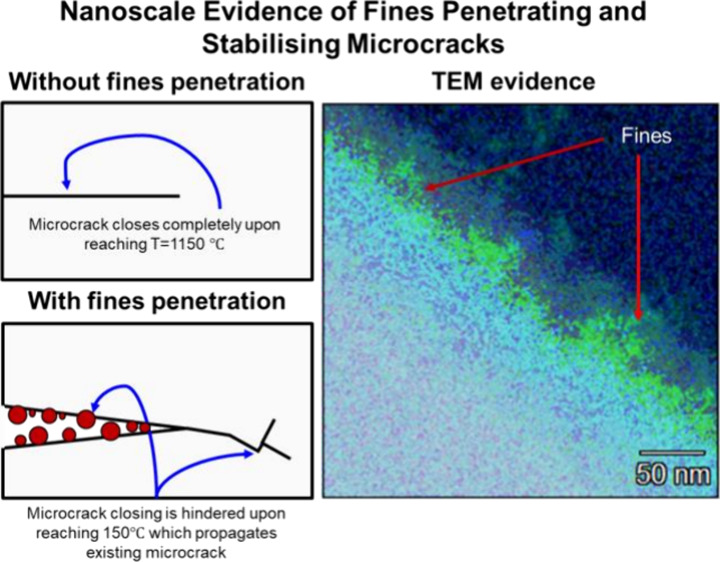

## 1 Introduction

Removal of air pollution remains one of the most significant challenges to improve human health and the environment, of which the predominant source is road transport. Strict emission standards have been imposed to save an estimated 34,700 premature deaths between 2027 and 2050 in many countries, including the EU.^1,2^ Monolithic substrates have emerged as the primary emission control catalyst media, enabling vehicle manufacturers to comply with emission standards. Substrates must exhibit excellent thermal durability under high-temperature conditions,^3^ with ceramics such as aluminum titanate (AT) gaining significant attention as a preferred material for construction, mainly attributed to their low thermal expansion, excellent thermal shock resistance, and high melting point. These desirable properties result from the inherent microcracks formed during production.^4,5^

Microcracks have a dynamic thermomechanical response, and a thorough understanding is critical to optimize durability. Clarke and Green^6^ reviewed various methods for characterizing microcracks, including direct and indirect techniques. Previous studies have established that dynamic high-temperature Young’s modulus measurements are a robust indirect way to characterize microcrack responses^5,7^ ; however, they were limited to uncoated materials and neglected the impact of coatings on the catalyst. As the literature shows that microcracks significantly contribute to the material’s durability, it becomes crucial to understand the influence of washcoat parameters on the thermomechanical responses of microcracks. Although microcracked materials have excellent durability, applying a washcoat to such materials presents challenges, such as inducing damage and cracks in the final manufactured catalyst.^8^

Optimization of washcoat parameters is essential since these directly impact the in-process performance of the coated substrates. Additionally, washcoats commonly have a bimodal particle size distribution (PSD) due to milling, producing submicron fines.^9^ Various parameters, such as PSD and viscosity, have been extensively studied with alumina being frequently used as the washcoating material; its subsequent impact on the monolith’s performance is commonly evaluated.^9–13^ However, these studies have not focused on the interactions between the fines resulting from the washcoating process and the microcracks in the substrate.

Significant progress has been made in integrating secondary phase particles within microcracked ceramic matrices, primarily to optimize their robustness and durability when used in high-temperature applications. Despite this innovation, the potential impact of fines is often overlooked. For example, Yoleva et al.^14^ investigated the addition of a mullite phase into AT and subsequently evaluated material properties. Similar studies investigated the effects of doping agents by modifying the secondary phases before sintering.^15–19^ The significant focus on material optimization in these investigations has resulted in a substantial gap in understanding the interaction between fines and microcracks, which is essential for enhancing the durability of microcracked ceramics. Montanaro et al.^20^ addressed the interaction between secondary phase particles and substrates, mainly regarding the soot generation process. They noted that the interaction between soot particles and microcracks initiated new phases and microcracks in cordierite. The focus on the influence of secondary phase particles on material durability identifies the need for this investigation to assess the impact of how fines from washcoats and microcracks interact.

The patent by Glasson et al.^8^ sheds some light, reporting penetration of microcracks by fines which cause local damage; however, no direct evidence was provided. In addition, the patent failed to provide details of the microscopy methodology used to image fines penetration and the reported dendrite structure formation within the microcracks. There are significant technical challenges in applying complex microscopy methods to this problem, which are not yet addressed. Furthermore, the assessment of Young’s modulus and damage parameters is central to exploring how fines affect microcrack responses, implying that changes in Young’s modulus are due to the formation of dendrite structures within microcracks arising from fines penetration. However, these claims were not backed by comprehensive observations. This motivated this research to investigate the underlying microcrack fine mechanisms and their implication on material performance. This study addresses this gap by overcoming the technical microscopy challenges not addressed in the patent, thereby building a comprehensive approach required to study such microstructural-level interactions.

Direct measurement methods, in conjugation with thermomechanical test methods such as the high-temperature impulse excitation technique (IET) and three-point bend test (3PBT), are required for a comprehensive understanding. In addition to scanning electron microscopy (SEM), advanced microscopy methods, such as (focused ion beam) FIB-SEM and transmission electron microscopy (TEM), offer convincing avenues for evaluating complex microstructural-level interactions. SEM can reveal microcracks within polycrystalline structures, though primarily limited to the material’s surface.^15,20–23^ FIB-SEM has gained increased attention for direct microstructural characterization in damage mechanics. For example, Yang et al.^24^ applied FIB-SEM to study microcracks generated within hard metals arising from a grinding process. In addition, an increasing number of studies have adopted FIB-SEM as a preparatory step for site-specific analysis and highlighted the efficacy of FIB in microscale sectioning for the precise excision and imaging of ceramic and biological samples.^25–28^ However, FIB-SEM must complement a more in-depth analytical method, such as TEM, to build a detailed understanding of the fines–microcrack interaction. Previously, TEM coupled with energy-dispersive X-ray spectroscopy (EDS) has been applied to study the impact of secondary phase particles, such as MgO, NiO, and ZrO_2_, within alumina matrices.^29^ Such techniques have elucidated the influence of secondary phase materials applied to substrates. However, these methods have not yet been applied to examine microcracked structures.

This study aims to provide the first direct nanoscale visualization of fines penetrating microcracks in coated microcracked catalysts. IET measurements are the primary tool to evaluate these interactions, supported by direct imaging methodology developed using SEM, FIB-SEM, and TEM. Through thermal cycling experiments, the study aims to determine if the influence of fines penetration is permanent, while strength testing (3PBT) quantifies the impact on the material strength.

## 2 Experimental Materials and Methods

### 2.1 Materials

For this investigation, the substrate of choice was a commercially sourced AT honeycomb filter, with specific sections of the AT honeycomb utilized as samples aligned with the methodology established in our previous study.^5^ For the coating process, commercially available alumina nanoparticles (Puralox) were utilized; this was crucial in formulating the slurry applied to the samples. This slurry was synthesized by combining the alumina nanoparticles with nitric acid and distilled water. The slurry was thoroughly examined to ensure a composition that would facilitate fines penetration into the microcracks of AT.

### 2.2 Experimental Method

#### 2.2.1 Synthesis and Characterization of Washcoat Fines

To achieve a bimodal PSD, the slurry consisting of alumina fines was synthesized using a controlled milling process. The commercial-grade alumina (Puralox) was combined with distilled water and nitric acid. This mixture was then milled for 240 min to produce a slurry containing a balanced distribution of fines and coarse particles. The slurry was then characterized to quantify the presence of fines through a two-step approach. The milled slurry was centrifuged at 3000 rpm for 30 min to separate the fines from the coarse fraction. The fines were then analyzed using dynamic light scattering (DLS) with a Zetasizer Nano to measure the PSD. The coarse particles were evaluated using the laser diffraction method using a Mastersizer 3000. The PSD results presented in Figure 1 are constructed by combining data from both these methods. Figure 1a shows the PSD of the synthesized alumina slurry, exhibiting a bimodal PSD with distinct peaks denoting fines and coarse particles. Figure 1b shows the result from the DLS method, resulting in a narrow distribution of fines and a D_50_ of 21.8 nm. These steps in synthesizing the slurry were critical to ensure the slurry met specifications comparable to typical commercial slurry formulations.

**Figure 1:**
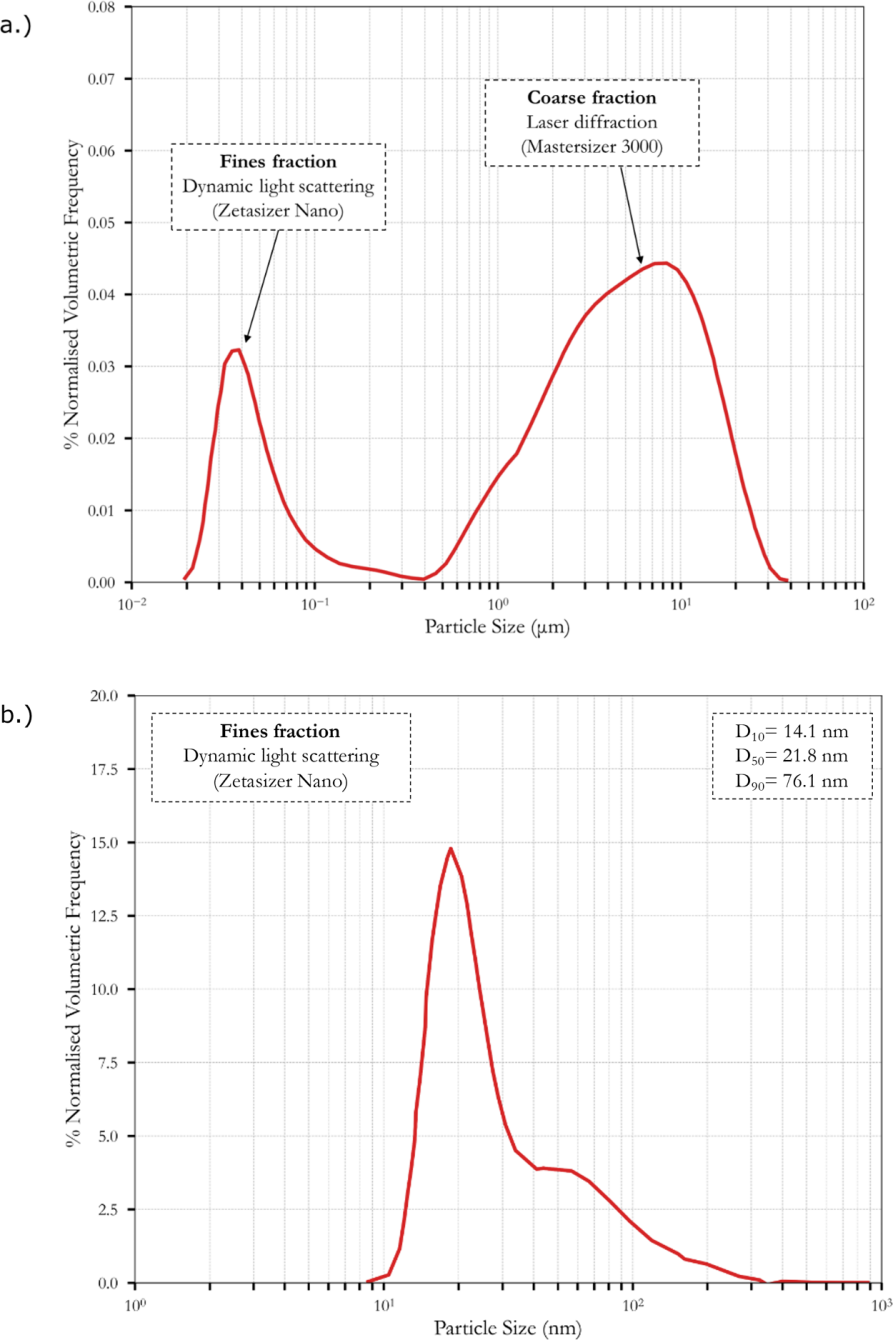
PSD of synthesized washcoat. (a) Volume frequency of particles' postmilling, comparing coarse (laser diffraction) and fines (dynamic light scattering) fractions. (b) PSD for fines fraction determined using DLS.

#### 2.2.2 Dip Coating Method

For the dip coating process, polymeric tape was used to protect the external surfaces of the uncoated AT samples. This control measure was implemented to achieve an internal-only wall coating. The ends of the samples were not covered by the tape to ensure that the slurry coated only the honeycomb channels. The taped AT sample was then immersed in a beaker containing 250 mL of the synthesized slurry. Via this immersion, the capillary forces allowed the slurry to rise through the channels. Each sample was submerged for 1 min and subsequently rotated and reimmersed from the opposite end, also for 1 min, to ensure complete coverage of the slurry in the honeycomb channels. An air gun was then used to remove any excess slurry from the sample channels, making this step critical in ensuring a homogeneous coating distribution.

It should be noted that as part of the dip coating procedure, the viscosity of the alumina slurry was repeatedly tested after coating five samples. This was done to ensure the consistency of the slurry was maintained for all coated AT samples because using the same slurry to coat all samples can cause dewatering of the slurry and a consequent increase in viscosity. No significant deviation in the viscosity was observed, and uniform coating and effective penetration of the fines into the substrate’s microcracks were obtained. This ensured that the thermomechanical performance of the applied wash coat was maintained across all samples.

After the coating process, the samples were dried for 24 h at 150 °C, mimicking a typical drying step in catalyst manufacturing. To achieve a higher loading, the immersion process and drying were repeated until the desired loading was reached. This method confirmed a high degree of repeatability and precision and is also supported by the previous works of Mogalicherla and Kunzru.^12,13,30^

#### 2.2.3 Dynamic High-Temperature Young’s Modulus Measurements

Building upon the methodology established in our previous study (Naudiyal et al.), IET was again utilized to measure the dynamic high-temperature Young’s modulus, thereby facilitating an analysis of the thermomechanical responses of microcracks in the AT material. However, the novelty provided by this study is the application of IET for characterizing microcrack responses subjected to the washcoating process, in particular, to understand the impact of fines penetration. A series of high-temperature experiments, including thermal cycling, were conducted within the IET furnace to explore the long-term effects of fines penetration into the microcracks. The coated AT samples underwent thermal cycling to 500 °C at a controlled heating rate of 5 °C/min for these experiments. The sample was then maintained at the peak operating temperature (500 °C) for 1 h and cooled to room temperature at a 4 °C/min cooling rate. Subsequently, the sample was allowed a significant relaxation period of 50 h at room temperature, enabling the microcracks to return to their initial state, as detailed in our previous work.^5^ For the thermal cycling experiments, this heating, dwelling, and cooling sequence was repeated across four cycles to investigate the thermomechanical behavior of the microcracks across repeated thermal exposures, ultimately to understand the long-term impact of microcrack–fines interaction.

Several vital steps were implemented for the thermal cycling experiments to mitigate potential damage to the material from repeated excitations. First, as the study focused on understanding fines penetration effects on microcracks, the peak operating temperatures were limited to 500 °C, and the calcination parameters, such as heating and cooling rates, were controlled. Other additional measures included increasing the interval between taps and reducing the tap intensity. Reducing the tap intensity was useful because applying the coating to the AT sample increased the material stiffness, which did not require vigorous excitation, which would otherwise have been essential for an uncoated AT sample. Finally, to further maintain the sample’s integrity, Young’s modulus measurements were deliberately omitted during the 50 h dwell periods to minimize damage from repeated tapping.

#### 2.2.4 Microscopy Analyses

SEM, FIB-SEM, and TEM were applied in this investigation to assess the complex microcrack–fines interaction and relate the direct observations made through these sophisticated analytical techniques to complement the observations of IET, serving as the direct evidence for the indirect insights of microcracks achieved from IET. The combination of microscopy methods provided unique insights into the changes at the microstructural level, particularly microcrack–fines interaction, resulting in a holistic view of the coating’s impact.

##### 2.2.4.1 SEM

SEM analysis was extended to assess the coating influence. This analysis included both coated and uncoated AT before and after the thermal cycle. Through this comparison, SEM provided key insights into the influence of the coating process and thermal treatment on the morphology of the microcracked surface of AT. This comparison at the microscopic level is critical in elucidating the complex microcrack–fines interaction. The SEM methodology utilized in this study aligns with the procedure highlighted in our previous study.^5^

##### 2.2.4.2 FIB-SEM

In this study, FIB-SEM was initially used as the primary tool for assessing the microcracks’ cross sections to understand the fines’ penetration inside them. However, the approach did not provide clear evidence of fines penetrating microcracks despite the high resolution and precision offered by FIB-SEM. Therefore, this investigation utilized FIB-SEM as a preparatory tool instead of a direct analytical method. This change in microscopy strategy was driven by the need for site-specific analysis in areas of the sample where the fines penetration into microcracks was highly likely. To identify the sites of interest, initial SEM images were utilized, highlighting SEM’s critical role in this study.

Producing the lamella structure for TEM analysis was laborious due to the number of steps involved. Figure 2 illustrates the various stages of lamella sample preparation. Figure 2a illustrates the initial surface of AT observed in FIB-SEM. The surface is characterized by a rough texture with visible microcracks and assorted debris, potentially from the material’s sample preparation or intrinsic nature. No fines are immediately detectable within the microcracks at this stage. A specific microcrack was targeted for milling; it was selected because it was covered by the coating, which was evident from the SEM result. Hence, it was highly anticipated that the fines from the coating would penetrate this microcrack. The carbon strip was then carefully deposited at the identified site for milling. Figure 2b illustrates a clean rectangular trench surrounding the microcrack, highlighting FIB-SEM’s capability to isolate the area of interest. This resulted in smooth and vertical milled walls, reflecting FIB’s accuracy. The image in Figure 2c displays a side view of a microcracked sample with the needle attached to it. Here, the microcrack cross-section is visible, and the depth and morphology of the crack are further highlighted. Additionally, the sample has been cleanly cut, and what is left behind is a well-defined structure that reveals the structure of the microcrack’s cross-section. Figure 2d illustrates that the microcracked lamella structure, representative of the microcrack cross-section, has been successfully attached to the copper grid. This was a product of precise milling on all sides and is a crucial preparation step for TEM imaging. Additionally, the ultrathin cross-section is beneficial for TEM in allowing high-resolution analysis and capturing microcrack–fines interactions.

**Figure 2:**
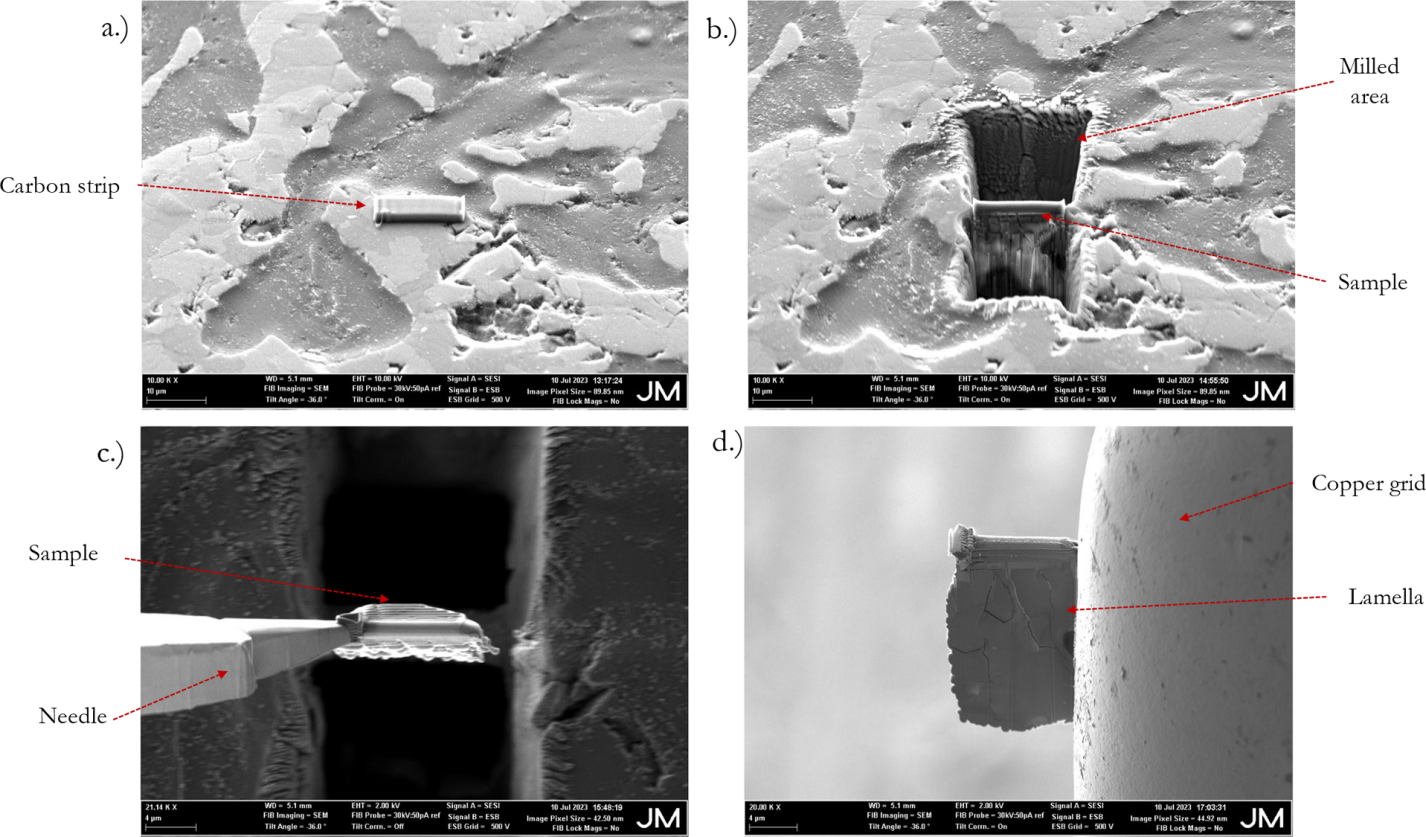
Lamella structure preparation for TEM using FIB-SEM for site-specific analysis. (a) Carbon strip deployed on a specific microcrack identified with coating. (b) Milled to obtain the cross-sectional sample. (c) Sample attached to the needle. (d) Obtained lamella attached to the copper grid for further processing and subsequent TEM analysis.

The capabilities of FIB-SEM are highlighted in Figure 2 for advanced sample preparation. This pragmatic approach ensured successful and detailed TEM analysis by carefully selecting specific areas of interest, especially considering the time-intensive nature of the FIB-SEM sample preparation. Although this methodology was laborious, it emerged as the most viable strategy for investigating the fines’ penetration into microcracks. This effective coupling between FIB and TEM also encourages researchers not only to limit the use of FIB-SEM to microstructural analysis but also to use it as a detailed-oriented preparation method to enable more focused investigations via TEM.

##### 2.2.4.3 TEM

Following the lamella preparation facilitated by FIB-SEM, the specimens were subjected to TEM analysis for an in-depth microstructural evaluation, particularly the cross sections of microcracks within the AT material. TEM analysis enabled the nanoscale examination of the microcracks and fines, hence offering unique insight into the interior of microcracks and the potential of fines penetrated within. In this study, TEM was coupled with EDS mapping, ensuring that the presence of fines within microcracks is accurately identified.

#### 2.2.5 Strength Testing

It was critical to evaluate the impact of fines penetration on the durability of AT; hence, strength testing was conducted. The flexural strength of the honeycomb AT samples was evaluated by using a three-point bend test setup (Instron). The samples for the strength test consisted of 18 cells in the width direction and 9 cells in the thickness direction. The preparation method for these samples adheres to the sample preparation procedure established in the previous investigation.^5^ One hundred defect-free rectangular honeycomb bars were produced following this established methodology.

The hundred samples were divided into four categories, each category with a minimum number of 25 samples, to allow meaningful comparisons. The resulting strength data was analyzed using a 2-parameter Weibull distribution analysis. The categories for strength analysis are highlighted in Table 1. The categories included uncoated AT material (class A) not subjected to thermal cycles, serving as a baseline for the inherent material strength. Uncoated AT samples (class B) underwent four thermal cycles to assess the effects of thermal cycling on the material strength. Coated AT samples with a 4% alumina loading were both dried only (class C) and subjected to drying and four thermal cycles (class D). This designed parametric approach helped analyze the impact of coating and thermal cycling on material durability. Figure 3 shows the 3PBT setup, where a span length of 50 mm and a load cell of 1 kN were utilized. Adopting a two-parameter Weibull distribution analysis allowed for determination of the strength for each class of samples, hence providing a meaningful comparison across the different conditions. This approach aimed to elucidate how the stiffness evolution of AT from its uncoated state through to postcoating and subsequent thermal cycling affects the material strength. The deviation in material stiffness observed among the uncoated, coated, and thermally cycled samples underpins the basis for categorizing the samples for strength testing, as shown in Figure 3, with the expectation that these variations would be reflected in the measured material strengths. Samples B and D, involving four thermal cycles, shown in Table 1, were prepared using a defined oven program identical to the thermal cycles conducted and described inside the IET furnace. However, due to the increased sample requirement for strength testing, the thermal cycling for these samples was conducted in a separate induction oven and not in the IET furnace. However, the heating mechanism of both furnaces was identical and used the same defined thermal profile as described in the IET thermal cycling experiments.

**Table 1:** AT Classes Used for Strength Testing

samples(*N* = 100)	description
A	uncoated (no thermal cycles)
B	uncoated (4 thermal cycles)
C	coated—dried only
D	coated—dried and 4 thermal cycles

**Figure 3:**
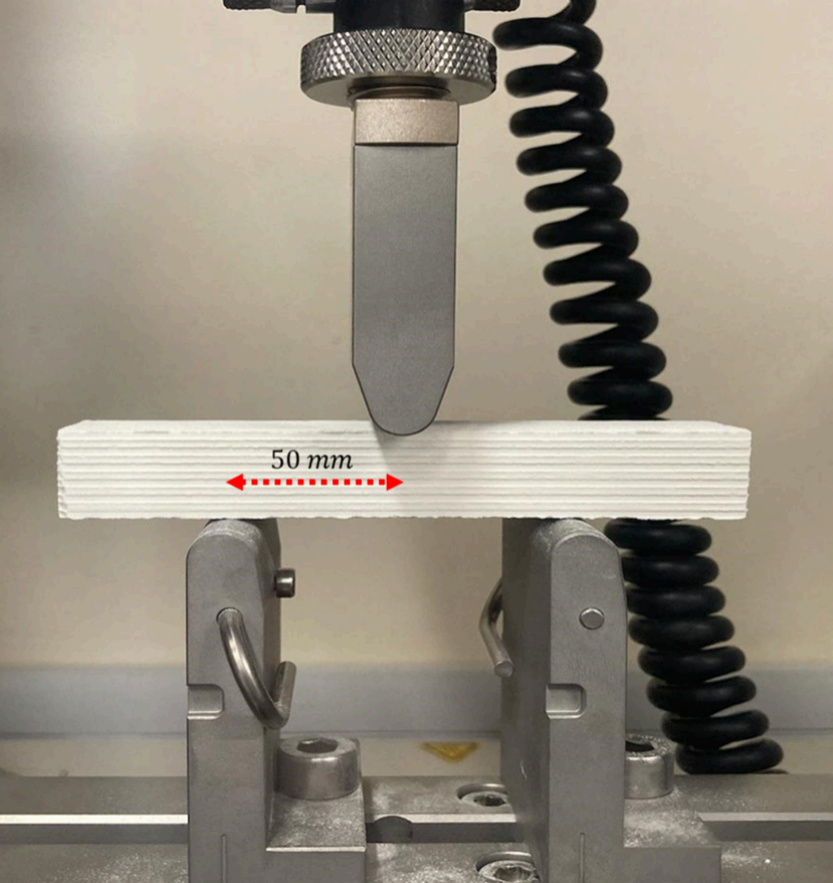
Three-point bend test setup for a AT honeycomb bar with a load span of 50 mm using a 1 kN load cell.

## 3 Results and Discussion

### 3.1 Effect of Washcoat Loading on Room-Temperature Young’s Modulus

The repeatability of the dip coating method was determined by evaluating changes in the room-temperature Young’s modulus of the AT samples with varying washcoat loadings. As illustrated in Figure 4, the average Young’s modulus for uncoated AT specimens was 1.26 ± 0.02 GPa. This value significantly increased with the application of washcoat, reaching 2.94 ± 0.02 GPa for a 4% loading and 3.76 ± 0.09 GPa for a 12% loading (loading indicating a percentage increase in weight due to washcoat application). It showed that increasing the washcoat loading enhanced material stiffness due to the increased material density resulting from solid deposition from the slurry onto the AT samples. The low standard deviations in the average Young’s modulus and washcoat loading reflected the high repeatability of the dip coating method. Although these results demonstrated an interaction between the washcoat and AT substrate, resulting in increased stiffness, they did not provide evidence of whether the washcoat particles penetrated the microcracks. Hence, the hypothesis remains central to this investigation, mainly if the fines from the washcoat have penetrated the microcracks. The increase in Young’s modulus observed is aligned with the literature, suggesting that material deposition increases stiffness. However, further analysis was required to determine if the root cause was due to fines penetrating microcracks or solely due to the surface-level coating–substrate interaction. This initial result sets the stage for subsequent SEM analysis, which aimed to further understand the underlying mechanisms that govern this increase in stiffness.

**Figure 4:**
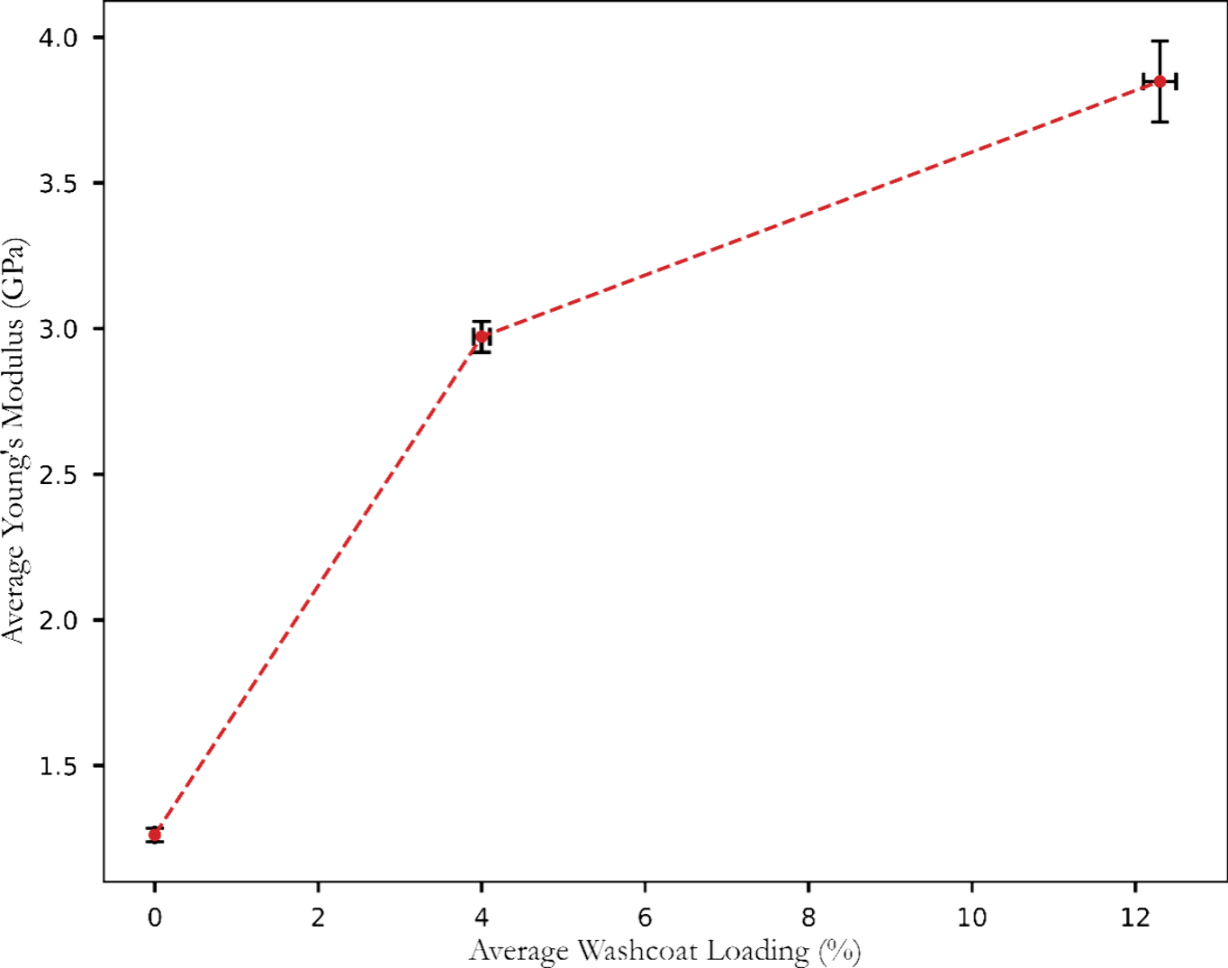
Effect of washcoat loading on the room-temperature Young’s modulus of AT bars.

### 3.2 SEM Analysis

SEM analysis is utilized to determine the location of the washcoat. Based on the PSD and slurry rheology used for coating, washcoat can be either on-wall (coating is on monolith channel webs) or in-wall (volumetric pore filling). SEM analysis in this work provided much further insight into the microstructural changes associated with varying washcoat loadings, mainly focusing on the microcrack–fines interactions. Figure 5 shows the SEM analysis of three AT samples required for comparative analysis, mainly to understand how varying washcoat loadings influence the microstructure of the microcracked AT. Figure 5 shows three sets of cross-sectional SEM images of honeycombs under varying magnification, 100 to 10 μm, presented left to right, respectively. Prior to this SEM analysis shown in Figure 5, the samples were embedded in resin, polished, and carbon-coated to ensure a representative image.

**Figure 5:**
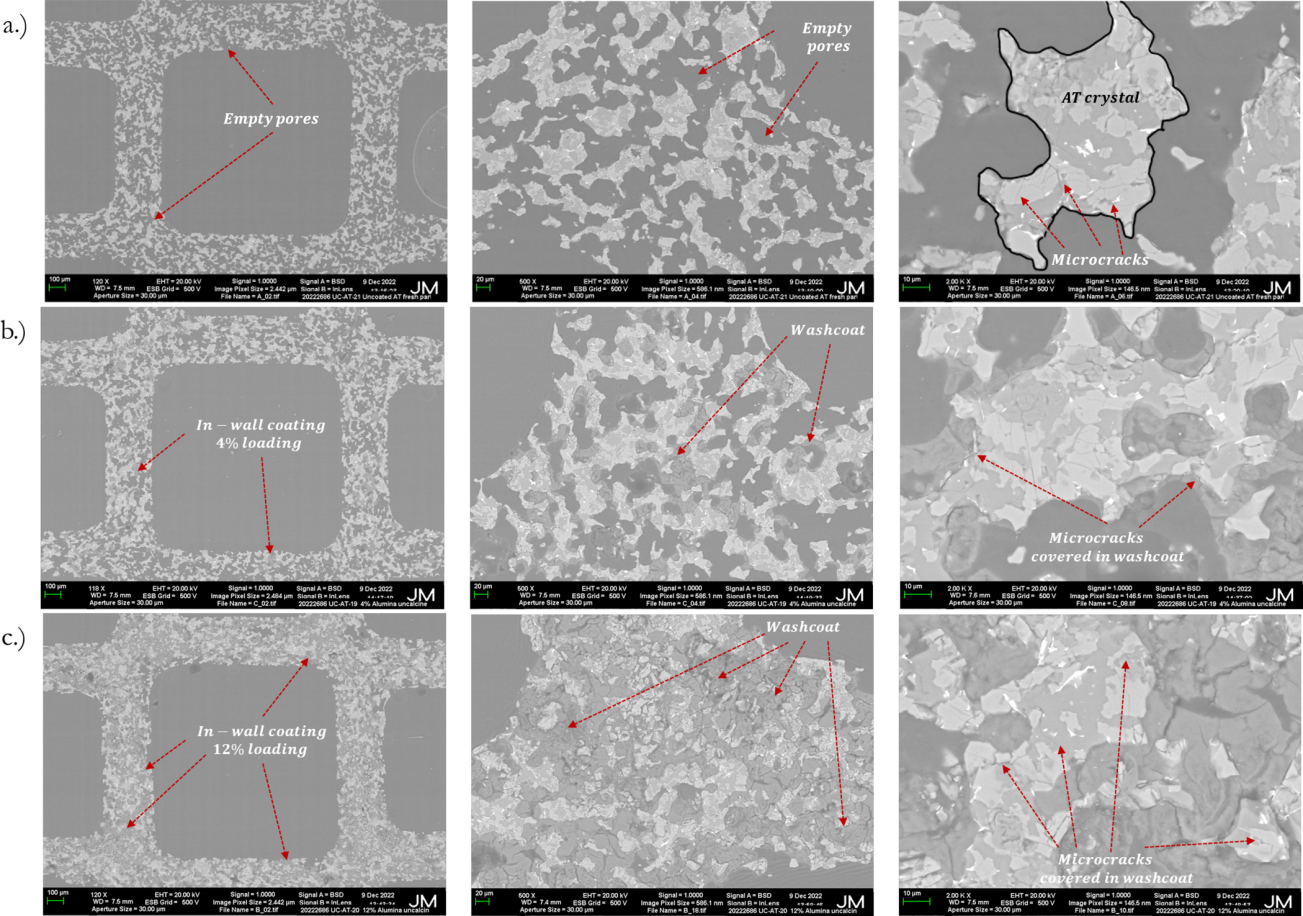
SEM analysis of the AT samples: (a) uncoated, (b) coated with 4% loading, and (c) coated with12% loading.

Figure 5a shows the uncoated AT, which is the bare substrate material showing the microstructure of AT when no secondary phase material, i.e., a coating, is present. The AT microstructure exhibited a smooth surface with pores; however, no coating-induced features were observed. Figure 5b shows the SEM image for coated AT at 4% loading, revealing a noticeable amount of coating on the cross section. The washcoat in Figure 5b partially filled the pores and microcracks compared to the uncoated AT surface. Figure 5c shows the SEM for coated AT with 12% washcoat loading. A denser coating is visible here, covering a more significant fraction of the cross-section. This increased coverage of the pores and microcracks further explains why a significantly higher Young’s modulus was observed for the coated AT at this loading.

Both coated samples in Figure 5b,c confirmed that the synthesized washcoat acts as an in-wall coating, effectively adhering to AT’s surface and highlighting regions where the washcoat has filled pores and covered microcracks. Although the SEM images in Figure 5 provided a detailed macroscopic view of the washcoat distribution, they did not again offer any conclusive evidence of the penetration of the fines into the microcracks. It showed that SEM analysis is limited to surface-level analysis and could not capture potential changes that could have occurred within the microcracks' interior structure. SEM failed to reveal the underlying microstructural changes associated with the significant variations observed in the room-temperature Young’s modulus once the samples were coated. It could be because such complex microstructural alterations occur beyond the surface level. Hence, our hypothesis that the fines penetrate inside the microcracks could be valid and could cause a significant increase in Young’s modulus. However, it could not be confirmed through SEM alone. While Figure 5 highlights that while SEM is a valuable tool for visualizing surface-level material morphology, it cannot sufficiently verify the depth of coating penetration or any internal changes to the microstructure. Furthermore, this justified the requirement for deeper analysis, such as TEM, ultimately to confirm fines penetration into microcracks.

### 3.3 High-Temperature IET

Following the results from room-temperature IET and SEM analyses (Figures 4 and 5), where the penetration of fines into microcracks could not be definitively confirmed, high-temperature IET experiments were conducted to characterize the dynamic responses of microcracks under thermal stress. Figure 6 presents the dynamic high-temperature Young’s modulus for uncoated AT samples and those coated with 4 and 12% washcoat loadings, serving as a critical method for indirectly assessing the penetration of fines into the microcracks and their effect on the material's thermomechanical properties under varying thermal conditions.

**Figure 6:**
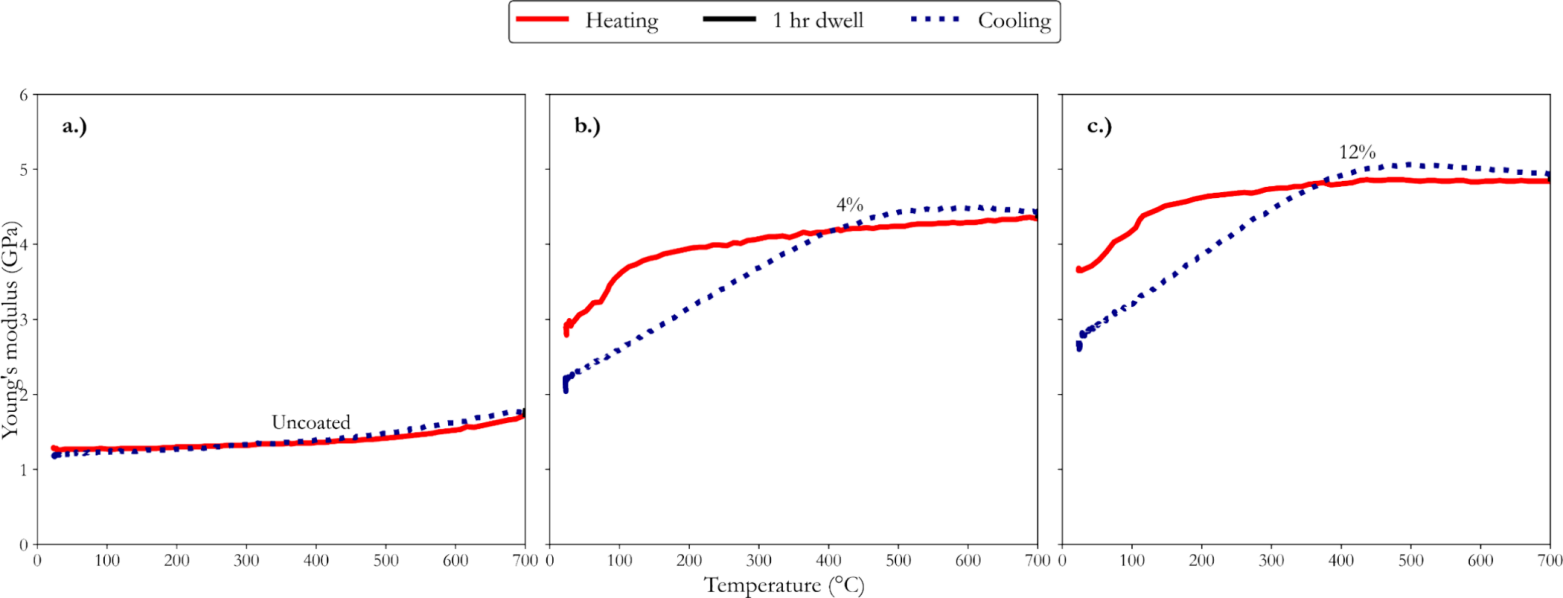
Thermomechanical responses of microcracks analyzed indirectly using high-temperature IET at various washcoat loadings (a) uncoated, (b) 4% loading, and (c) 12% loading. All investigated at a constant heating rate of 5 °C min^–1^, a dwell period of 1 h, and a cooling rate of 4 °C min^–1^.

Previous investigations demonstrated that Young’s modulus increased before plateauing in bare substrates at approximately 1100 °C due to the contact of microcrack surfaces, which rendered the material porous.^5^ However, when a coating is applied, similar phenomena are observed at lower temperatures in the current investigation. As shown in Figure 6, Young’s modulus for the uncoated AT samples remained relatively stable across the entire temperature range during heating and cooling cycles. This stable behavior suggested that the microcracks within the uncoated AT did not exhibit thermal responses below 500 °C. This observation was as expected and identical to our previous finding, indicating that microcrack closing or opening at temperatures lower than 800 °C results in minimal hysteresis.^5^ In contrast, the coated samples with 4 and 12% washcoat loadings showed significant variations in Young’s modulus throughout the thermal cycles, providing indirect evidence that fines have penetrated the microcracks.

In Figure 6, during the heating cycle, a significant increase in Young’s modulus was observed up to approximately 150 °C for both coated samples. Stiffness increases from room temperature to 150 °C may indicate partial closing of microcracks due to the interaction with fines. The plateau observed from 150 to 500 °C indicated that further closing or interaction is inhibited, likely due to the fines becoming lodged within the microcracks, thereby preventing additional closure as the temperature of the material increases. During the 1 h dwell period, a slight increase in material stiffness was noted, particularly in the 12% washcoat loading sample, indicating a delayed microcrack closing response.^5^ This behavior implied that microcracks respond to thermal stress by partially closing or interacting with the fines.

The cooling cycle also indicated that the fines penetrated the microcracks in AT. As the temperature decreased from 500 °C to approximately 400 °C, Young’s modulus initially exceeded the values recorded during the heating cycle, with an intersection point observed at around 400 °C for the 4% loading and 380 °C for the 12% loading. This intersection point is critical, as it marked the temperature at which the stiffness declined in the cooling cycle and diverged from the heating cycle. The modulus progressively decreased below these intersection points, and the rate of change in Young’s modulus appeared nearly identical for both samples, with the cooling curves running almost parallel below 400 °C. This significant reduction in Young’s modulus upon cooling to room temperature, particularly in the coated samples, suggested that the microcracks may have reopened and that new microcracks have formed due to thermal cycling. The substantial decrease in stiffness indicates microstructural changes that are likely exacerbated by the presence of fine particles within the microcracks. The inability of the material to recover to its initial stiffness after the cooling cycle suggested that the penetrated fines contributed to the enlargement of the existing microcracks or perhaps the formation of new ones, supported by the dramatic reduction in Young’s modulus observed in both coated samples.

In Figure 6, the high-temperature IET results provided compelling evidence that fines penetrated the microcracks, affecting the AT’s stiffness. This penetration appeared to be responsible for the observed changes in stiffness during thermal cycling, including the notable reduction in the room-temperature stiffness upon cooling. The increased hysteresis behavior for coated AT occurred at significantly lower operating temperatures than uncoated AT, which occurs at much higher temperatures, as observed in our previous study.^5^ These findings further support the hypothesis of fines modifying the microcrack responses during thermal treatment.

### 3.4 Hypothesis of Microcrack-Fines Interactions

The open-loop hysteresis in Young’s modulus of the coated structure indicated some microstructural changes in the filter material. To understand this phenomenon, a hypothesis was formulated regarding the role of fines in modifying microcrack responses during thermal treatment; all findings from room-temperature IET, SEM, and high-temperature IET were integrated into formulating this hypothesis. The remainder of this study is devoted to testing the proposed hypothesis on the role of fines expressed in Figure 8 using TEM analysis, IET thermal cycling analysis, and strength testing—designed to offer further evidence to support or challenge this hypothesis.

Figure 7 shows the proposed hypothesis presented in a schematic. Starting with the uncoated AT material, the microcracks remain open at room temperature, close upon heating, and ultimately heal at the complete microcrack closing temperature (*T*_H_). The schematic in Figure 7 reflects that in the cooling cycle, microcracks reopen and return to their initial state, as shown in our previous study.^5^ However, this study focuses on the coating, and particularly the fines, hence the second part of Figure 7 focuses on the first thermal cycle of coated AT. The fines were hypothesized to penetrate the microcracks and prevent complete closure. During the first thermal cycle displayed in Figure 6 and now reflected through the schematic in Figure 7, as the temperature is increased, fines interact with microcracks; this hinders complete closing and results in instant plateauing in the material stiffness around *T*_P_ = 150 °C. Continued high-temperature exposure leads to the propagation of the existing microcracks due to the coupled effect of thermal stress due to differential thermal expansion of alumina and aluminum titanate and obstruction due to fines. Figure 7 hypothesizes the role of fines in subsequent thermal cycles, forecasting the microcrack responses in the second and *N* number of cycles. We, therefore, hypothesize that further microcrack propagation in the subsequent thermal cycles will occur due to hindered closing due to fines. This could potentially lead to macrocrack formation as the sample undergoes repeated thermal cycling. As a result of the coupled thermal fatigue and persistent interaction between fines and microcracks, the material microstructure could be significantly damaged, reducing the strength over time.

**Figure 7:**
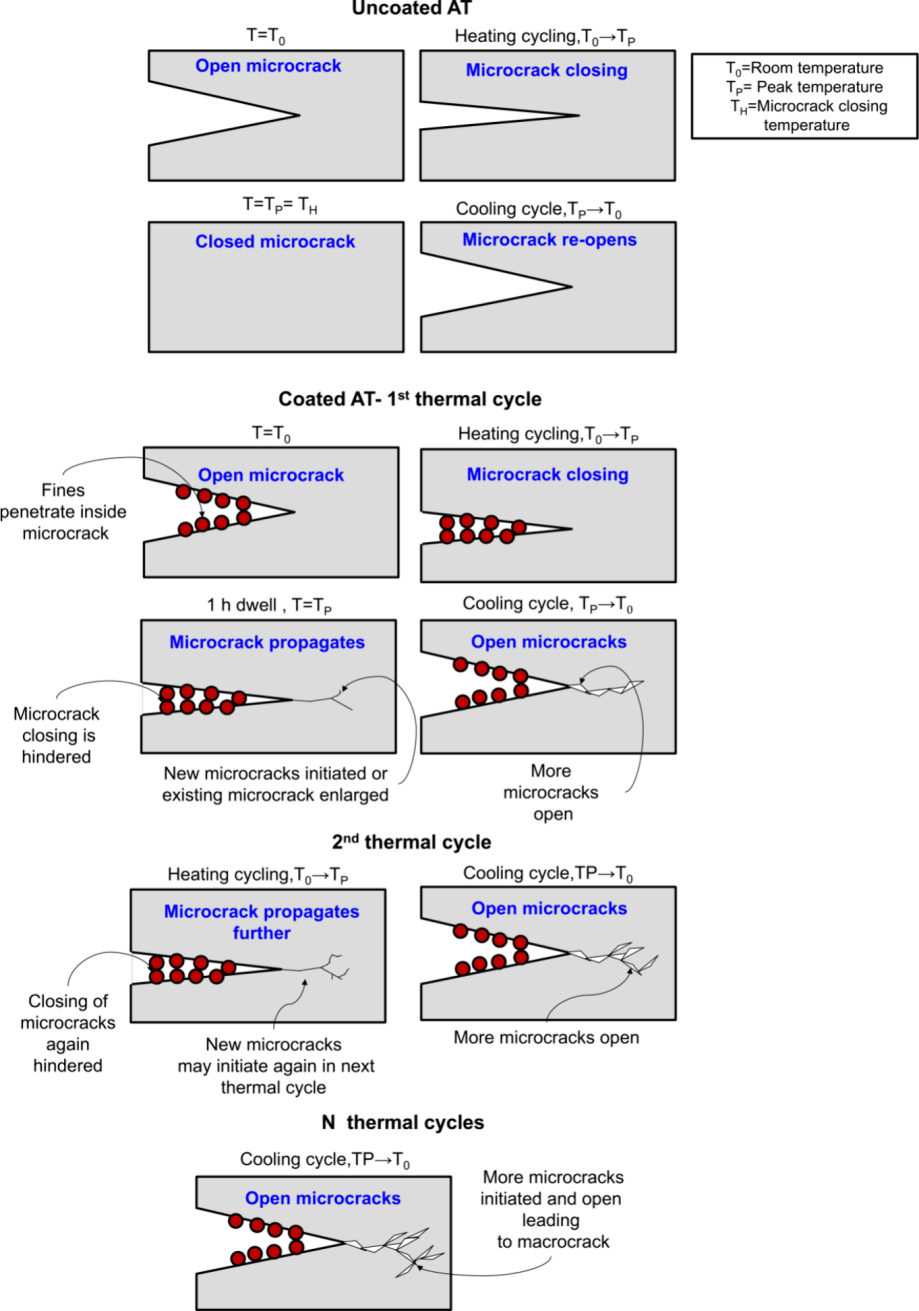
Hypothesis of fines from washcoat penetrating microcracks and the predicted consequences on the microcrack as a function of thermal cycles.

### 3.5 TEM Analysis: Direct Evidence of Fines Penetration

Figure 8 shows a series of images from the TEM analysis to investigate microcrack–fines penetration. The images show clear cross-sectional views of the microcracks in AT to provide a comprehensive assessment of the interaction between fines and microcracks, which was impossible through SEM and FIB-SEM alone. Additionally, the images are displayed at different magnifications in Figure 8 to provide a holistic view of fines interaction with microcracks.

**Figure 8:**
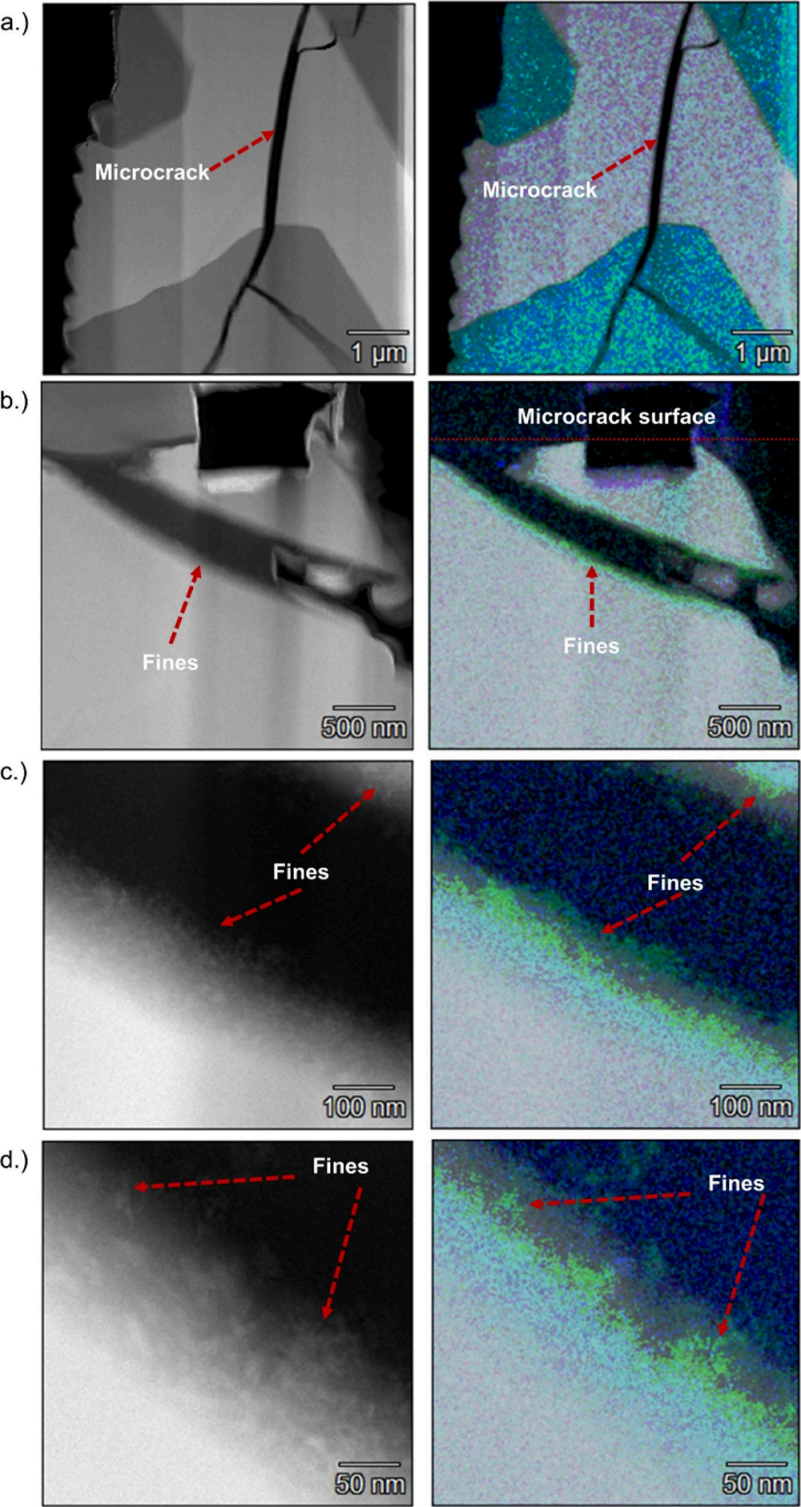
TEM image and EDS map of (a) uncoated (1 μm) AT, (b) coated AT (500 nm), (c) coated AT 100 nm, and (d) coated AT 50 nm. In the EDS maps, the green color corresponds to alumina fines and white for AT.

Figure 8a shows the uncoated AT, showing the inherent microcracks that allow a fair comparison to its counterpart. The microcracks observed here appear clean, with no evidence of external material penetration. The EDS analysis (right TEM image) also confirms that no alumina particles (green) are present as the AT material (white) is uncoated. Figure 8b shows the TEM result for a coated AT sample, revealing that the fines were deposited along the microcrack edges. This provided the first direct evidence that the fines from a washcoat penetrate the microcracks during the coating process. The EDS TEM image (right) confirms the presence of alumina fines (green) inside the microcrack, with the microcrack surface highlighted. Figure 8c,d provides an extremely high magnification view, 100 and 50 nm, respectively, of the coated microcracked AT. The fines appear more distinct inside the microcracks. Both TEM images provided direct visual confirmation of fines penetrating microcracks. Figure 8d, in particular with the extreme close-up view, shows how the fines from the coating adhere to the inner surfaces of microcracks. The fines were observed to form a film-like deposition onto the inner surfaces of microcracks, which is the reason for the significant change in the thermomechanical behavior of AT under high-temperature conditions, as evident through high-temperature IET experiments. The TEM analysis illustrated in Figure 8 supported the hypothesis that fines penetrate the microcracks. It provided direct evidence that the microstructure of AT has fundamentally changed. The results from the TEM analysis are also critical in providing direct evidence, which aligns with the indirect observations made using the result from high-temperature IET, which also showed that fines penetrated microcracks based on changes in the dynamic modulus during the thermal cycling.

The hypothesis that fines penetrate microcracks was validated through the findings of TEM shown in Figure 8, which provided conclusive evidence that fines appear between the inner surfaces of the microcracks, preventing them from closing at high temperatures. The notable observation highlighted from Figure 8 is that the fines penetrate the microcracks and significantly modify their thermomechanical responses, leading to altered material stiffness, as reflected in the high-temperature IET result.

### 3.6 Long-Term Impacts of Fines Penetration

#### 3.6.1 Effect on Material Stiffness

Figure 9 shows the dynamic Young’s modulus of AT across multiple thermal cycles, highlighting the long-term effects on the microcracks due to fines penetration. For completeness, the 4 and 12% washcoat loading data are included. Again, this was compared to the initial hypothesis regarding particle penetration’s long-term impacts on AT’s thermomechanical properties.

**Figure 9:**
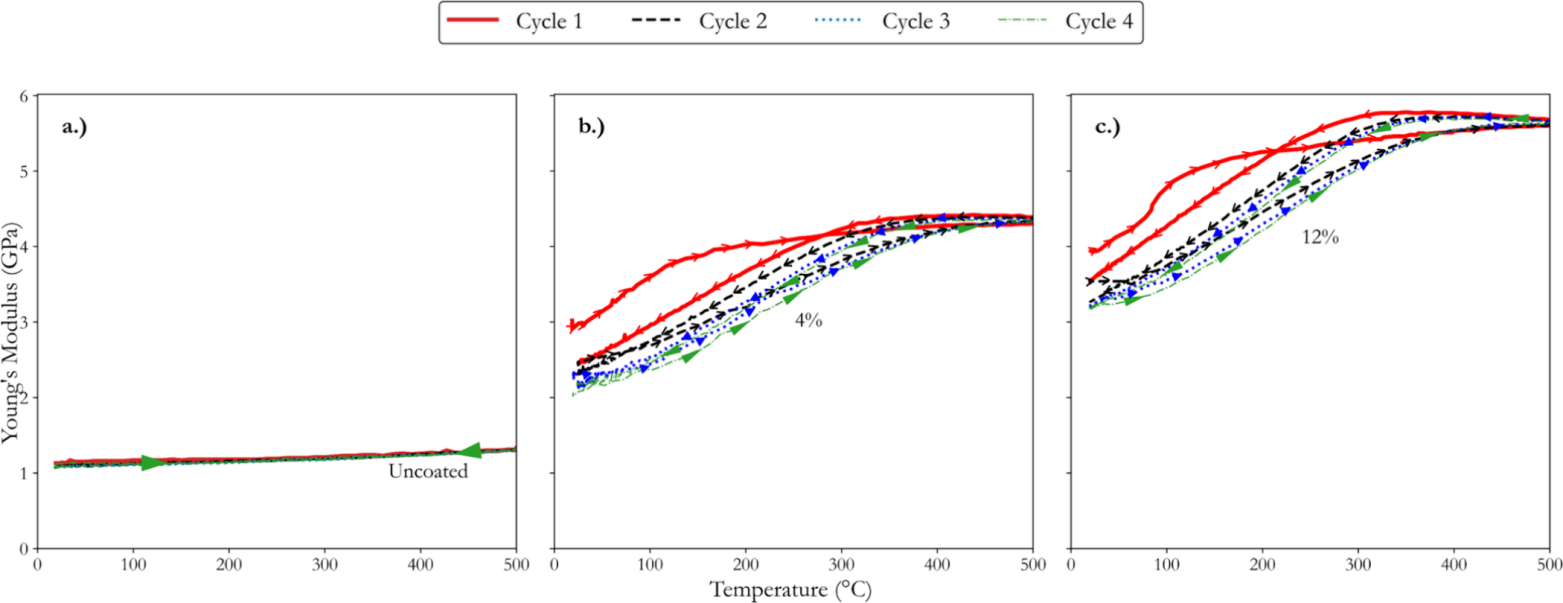
Investigating the long-term impact of coating and thermal cycling on the dynamic Young’s modulus of AT at various washcoat loadings: (a) uncoated, (b) 4% loading, and (c) 12% loading.

For the uncoated sample, Figure 9a shows minimal changes in Young’s modulus across the four thermal cycles, showing that the impact of thermal cycling is negligible in the absence of a washcoat for temperatures up to 500 °C. However, for the coated AT samples, 4 and 12% loading shown in Figure 9b,c, respectively, a significantly contrasting behavior is observed in comparison to the behavior of the uncoated material. For both these samples, a significant increase in Young’s modulus was observed during the first thermal cycle, aligning with the hypothesis that fines penetrated microcracks, reinforced the material structure, and enhanced the material stiffness.

As thermal cycling continues, the rate of change in Young’s modulus decreases with each successive cycle, resulting in a decreased hysteresis in the Young’s modulus. There was a slight further decrease in stiffness hysteresis from cycle 2 to cycle 3. However, no significant change in the hysteresis was observed from cycle 3 to cycle 4. Therefore, by the fourth cycle, the stiffness for the 4 and 12% loading samples converged, indicating that the microcrack responses reached a quasi-equilibrium state. The stabilization in stiffness post-third cycle suggested that although microcracks were initially enlarged in the first, second, and third thermal cycles due to the fines penetration, new microcracks were created post-third cycle. Additionally, it showed that the initial rate of microcrack propagation and any new microcrack formation due to enlargement slowed down considerably.

The observations made in Figure 9 supported our hypothesis that the penetrations of fines impact microcrack behavior during the thermal cycle. However, the influence of fines diminishes after only three thermal cycles. However, another perspective is that fines have permanently altered the high-temperature stiffness behavior of AT in comparison to its initial uncoated response. The findings supporting our hypothesis are that initially, fines hinder microcrack closure, as shown by increased AT stiffness, which is particularly evident in the first cycle, showing microcrack propagation. However, contrary to our initial expectation that continuous cycling would lead to a macrocrack due to anticipated ongoing microcrack propagation, the observations show that the material reached a stabilized state after three cycles. This deviation from our original hypothesis showed that the penetrated microcracks, in essence, reinforced the microcracks and prevented further propagation; hence, macrocrack development was averted. The microcrack responses became consistent, and hence, no further damage was accumulated in the material.

Overall, the findings highlighted in Figure 9 show that although fines resulted in increased microcracked density initially, in the long term, they did not result in unstabilized Young’s modulus behavior.

#### 3.6.2 Effect on Material Strength

Finally, to understand the impact of fines penetration, strength analysis was conducted to evaluate the material durability. Table 2 shows the results of the strength analysis, including the Weibull modulus, scale parameter, and mean strength. To achieve a holistic understanding of penetrated fines, the sample classes included both uncoated and coated AT, including coated samples subjected to four thermal cycles. This comprehensive strength testing aimed to integrate the findings from high-temperature IET experiments, TEM, and thermal cycling IET.

**Table 2:** Effect of Washcoat Loading and Thermal Cycling on the Material Strength

sample	Weibull modulus (95% CI)	Weibull scale parameter (95% CI)	mean strength (MPa) (95% CI)
uncoated	13.5 ± 2.85	1.31 ± 0.04	1.26 ± 0.03
coated—4% loading	11.8 ± 2.57	1.59 ± 0.05	1.52 ± 0.06
uncoated—4 cycles	12.1 ± 3.89	1.30 ± 0.04	1.25 ± 0.05
coated—4 cycles	10.4 ± 2.14	1.41 ± 0.05	1.35 ± 0.05

For the uncoated AT samples shown in Table 2, a Weibull modulus of 13.5 ± 2.85 and a mean strength of 1.26 ± 0.03 MPa were determined, serving as a baseline for comparison to the other classes. The result for the uncoated material showed a high reliability and uniformity in strength distribution. For the coated AT samples not subjected to any thermal cycles, the Weibull modulus decreased to 11.8 ± 2.57, indicating higher variability in strength. However, the washcoat enhanced the material’s overall strength despite introducing increased heterogeneity, as evident by the higher mean strength value, 1.5 ± 0.06 MPa. It showed that the washcoat reinforced the microstructure due to an increase in the density or decrease in porosity and fines penetrating the microcracks, enhancing material strength. This observation also aligned with the IET result and supported the proposed hypothesis.

The observations for the uncoated AT samples subjected to four thermal cycles showed a slight reduction in the Weibull modulus to 12.1 ± 3.89. This reduction could be due to damage accumulation resulting from thermal stress. Notably, no significant change in the mean strength was observed, remaining at 1.25 ± 0.05 MPa, highlighting that microcracks do not significantly evolve under thermal cycling without a washcoat. This observation was also accurate for the IET thermal cycling result, where no change in dynamic stiffness was observed for uncoated AT. On the other hand, the coated AT samples subjected to four thermal cycles showed the most significant decrease in the Weibull modulus to 10.4 ± 2.14, indicating the most significant variability in the strength across the four sample classes. This significant behavior change could be due to the combined effects of fines penetrating inside microcracks and subsequently undergoing four thermal cycles. The significant finding from Table 2 was that the mean strength of the coated sample remained significantly higher than all uncoated classes of samples despite continuous thermal exposure. The permanent increase in the material strength is advantageous for high-temperature applications. Interestingly, this increased strength also builds upon the proposed hypothesis that introducing a washcoat which penetrates the microcracks not only results in stabilized thermomechanical responses post-thermal cycling but also results in increased material durability by stabilizing microcracks and preventing significant degradation of the material over time.

The critical insights gained from the strength analysis in Table 2 underscored the effect of critical microcrack–fines interaction on the material strength by evaluating the Weibull modulus and mean strength. The strength analysis presented also provides another critical evidence for confirming that fines penetrated the microcracks and hence enforced the microstructure, resulting in higher mean strength for coated AT before and after thermal cycling. This evidence, using the strength data, also further backs the indirect and direct evidence provided through IET and TEM analyses, respectively, where both confirmed fines penetration. The strength analysis of the penetrated microcracked AT samples further supports that fines introduce heterogeneity in AT, as previously shown by significant changes in dynamic stiffness observed in thermal cycling.

Overall, the strength results presented in Table 2 built an additional critical understanding of the impact of penetrated fines when coupled with previous dynamic Young’s modulus data to provide a holistic perspective. The observations further built on the hypothesis that finely penetrated samples displayed a significant increase in strength even after repeated thermal cycling despite increased variability in strength.

## 4 Conclusions

This study presents the first direct nanoscale evidence of washcoat fines penetrating microcracks in a coated catalyst substrate, revealing their dynamic influence on material properties during thermal cycling. The fines penetrated microcracks, resulting in a significant reduction in the degree of damage to the material. Upon thermal cycling, the damage in the coated material increases due to the enlargement of penetrated microcracks, showing a detrimental impact of the penetration initially. However, after three thermal cycles, the damage stabilizes, and the final coated material possesses significantly higher stiffness and strength, showing that the fines penetration is ultimately beneficial.

IET provided indirect evidence of fines from the washcoat penetrating microcracks. FIB-SEM was helpful for site-specific sample preparation, with the subsequent TEM analysis providing direct evidence of this phenomenon. Fines penetrating microcracks resulted in significant stiffness enhancement in AT due to fines filling and reinforcing the microcracks. Thermal cycling initially led to microcrack enlargement and the initiation of new microcracks, as confirmed by strength testing. However, the damage stabilized after three cycles. The evolution of microcrack enlargement and initiation can be effectively tracked using IET, as reflected in the stiffness curves observed during thermal cycling. In strength testing, the stabilized state of the penetrated microcrack samples was reflected by the permanently higher mean strength than their uncoated counterparts, even after four thermal cycles.
